# *In situ* mapping of iron oxidation in laminar metal fuel flames using X-ray absorption spectroscopy

**DOI:** 10.1107/S1600577526005904

**Published:** 2026-07-15

**Authors:** Lukas Braun, Fabian P. Hagen, Michal Fedoryk, Malte Seitz, Björn Stelzner, Dimosthenis Trimis, Jan-Dierk Grunwaldt, Dmitry E. Doronkin

**Affiliations:** a Karlsruhe Institute of Technology Institute for Chemical Technology and Polymer Chemistry (ITCP) Engesserstr. 20 76131Karlsruhe Germany; b Karlsruhe Institute of Technology Institute of Catalysis Research and Technology (IKFT) Hermann-von-Helmholtz-Platz 1 76344Karlsruhe Germany; c Karlsruhe Institute of Technology Engler-Bunte-Institute, Combustion Technology Engler-Bunte-Ring 7 76131Karlsruhe Germany; ESRF – The European Synchrotron, France

**Keywords:** X-ray absorption spectroscopy, oxidation state mapping, metal flames, energy storage

## Abstract

Metal fuels such as iron are promising carbon-free energy carriers for a sustainable energy system, where energy release occurs via combustion in metal flames. Apart from the design of the appropriate burners, another challenge is to control the oxidation process and gain direct insight into the kinetics and the mechanism of metal oxidation in flames. Optimizing the combustion process to release the stored energy is crucial, but *in situ* analysis of metal flames remains a real chemical engineering and physical chemistry challenge. Here, we demonstrate an *in situ* approach with synchrotron X-ray radiation allowing identification and quantification of iron (oxide) phases during the combustion process that is adaptable for various combustion modes and conditions. Using quick scanning X-ray absorption spectroscopy, we were able to track the oxidation state of iron and thus the structure along the visual flame cross-section. Strong gradients in oxidation state and phase composition across and beyond the flame front were found. The flame front determined by visualization with an optical camera corresponds to the formation of FeO, while the main combustion product Fe_3_O_4_ was mainly formed outside the visible flame zone.

## Abstract 2

For the first time an iron metal flame has been mapped by *in situ*X-ray absorption spectroscopy. Strong gradients in oxidation state and phase composition across the metallic core and the flame front were found with FeO peaking at the visible flame front and Fe_3_O_4_ forming further beyond.

## 1. Introduction

The combustion of the widely available fossil fuels for energy production purposes and industrial use has been a major driving force for our economic growth, but also one of the main contributors to anthropogenic global warming (Höök & Tang, 2013 ▸). Therefore, a transition to sustainable and carbon-neutral energy systems is critical to mitigate climate change and ensure long-term energy security. In this context, the first step ideally could be to replace coal with metal fuel powders. Iron and its oxides have emerged as promising carbon-free energy carriers within a closed circular economy. This metal fuel has several advantages compared with other energy carriers (H_2_, batteries, methanol, *etc*.), foremost the high volumetric energy density, high availability of iron and its oxides, their low toxicity, possible decentralization and ease of transportation (Janicka *et al.*, 2023 ▸; Debiagi *et al.*, 2022 ▸; Neumann *et al.*, 2023 ▸). Further, due to its similar energy density (lower heating value, LHV, of Fe 57.9 GJ m^−3^ versus 30–57 GJ m^−3^ for coal) and combustion characteristics compared with coal, iron powder combustion could take place in existing, retrofitted coal-fired power plants, where the resulting iron oxides, unlike CO_2_, can be captured by conventional ash filters (Lett & Ruppel, 2004 ▸; Debiagi *et al.*, 2022 ▸). This approach exploits iron powder as a carbon-free replacement for coal, in line with global decarbonization targets, while repurposing existing infrastructure (Janicka *et al.*, 2023 ▸; Debiagi *et al.*, 2022 ▸). Green hydrogen produced from renewable sources, such as wind and solar power, can be used as a reducing agent for iron oxide particles to metallic iron that can then serve as metal fuel (Turner, 1999 ▸; Song *et al.*, 2022 ▸; Delucchi & Jacobson, 2011 ▸). The reduction process would be conducted in sun- and wind-rich regions, where the renewable energy production is the most efficient. Afterwards, the reduced iron can then be transported to a region with high energy demand, where the combustion step would take place to release the energy. Afterwards the oxidized iron is transported back, closing the cycle.

For an efficient usage of the stored energy in iron and a clean combustion, a better understanding of iron oxide reduction and processes during oxidation of Fe^0^ are needed. The reduction of iron oxides has been acquiring increasing interest in the recent years as a potential hydrogen storage process, while it has also been studied intensively due to its importance for the steel industry and industrial catalysis (Haber-Bosch process and Fischer–Tropsch synthesis) (Patlolla *et al.*, 2012 ▸; Braun *et al.*, 2024 ▸; Hacker *et al.*, 1998 ▸; Li *et al.*, 2002 ▸; Pernicone & Traina, 1979 ▸). The evolution of iron oxide phases was lately investigated with *in situ* synchrotron X-ray absorption spectroscopy (XAS)/X-ray diffraction (XRD) measurements and thermogravimetric analysis (TGA) during temperature-programmed reduction in small-scale fixed bed plug flow reactors or, respectively, crucibles in the case of TGA. It was found that the reduction starts at temperatures above 400°C, transitioning through different oxidation states via a core-shell mechanism. A micrometre-sized shell of metallic iron (Fe^0^) forms directly at the beginning on the surface of the hematite (α-Fe_2_O_3_) particles. This hinders the diffusion of H_2_O formed in the core regions due to the lower porosity of the iron shell. This leads to the formation of a magnetite (Fe_3_O_4_) intermediate layer around the core at temperatures between 500°C and 600°C. In case of fast heating ramps, the reduction is not complete below 600°C due to being limited by H_2_O diffusion and at this temperature wüstite (FeO) becomes thermodynamically stable, which leads to the generation of a wüstite (FeO) core/iron (Fe^0^) shell particles. In the final step the remaining wüstite (FeO) is reduced to metallic iron (Fe^0^) (Braun *et al.*, 2024 ▸; Yüzbasi *et al.*, 2018 ▸; Jozwiak *et al.*, 2007 ▸).

While the high-temperature oxidation of single micrometre-sized iron particles can be investigated in single particle experiments, the study of reaction kinetics in particle ensembles, including particle–particle interactions as encountered in realistic combustion environments, requires the use of conical iron dust flames. A Bunsen burner configuration enables the stabilization of self-sustained, axisymmetric, laminar iron dust flames under well defined boundary conditions. A homogeneous, steady-state aerosol of micrometre-sized iron particles is generated, forming an ignitable mixture of particles in an oxidizing gas. Although such laminar flames are readily accessible for probing, the *in situ* analysis of iron oxide phase composition remains challenging. Consequently, phase analysis typically relies on extractive particle sampling followed by *ex situ* characterization, which may perturb the flow field, flame structure and reaction kinetics. Sampling from an ongoing reaction typically involves the insertion of a probe into the flame to collect the particles, either passively or by extracting them from the reaction mixture and quenching them immediately for the subsequent *ex situ* analysis with methods such as, for example, X-ray diffraction, electron microscopy or Mössbauer spectroscopy (Fedoryk *et al.*, 2023 ▸; Buchheiser *et al.*, 2023 ▸; Chen & Yeun, 2003 ▸; Bertrand *et al.*, 2010 ▸).

Experimental investigations of iron–air flames to this date have mostly focused on identifying laminar burning velocities, flame stability or flame temperature. For further modelling and optimization of the burning process, deeper understanding of the phase evolution of iron oxides during the oxidation process is required, which has not yet been reported for metal flames under conditions that are relevant for industrial applications. Removing (sampling) the powder from the process for *ex situ* analysis inevitably alters conditions, such as temperature, pressure and gas environment, which may induce phase transformations. Thus, *ex situ* methods cannot provide definitive proof of the species present at a given time in the given location within the flame. Process models validated based on *ex situ* data may struggle to reproduce the actual process. However, *in situ* characterization of oxide phase distributions in metal flames is a significant technical challenge that, to date, has not yet been solved. Only very few studies in related research areas have attempted to address this issue, and synchrotron radiation plays a key role here (Beaucage *et al.*, 2004 ▸). For example, Camenzind *et al.* (2008 ▸) observed the formation of nanostructured silica particles in diffusion flames via small-angle X-ray scattering (SAXS). They were able to monitor the aggregate formation and particle density, but this technique did not provide distinct information about the oxidation state, or the phases present at the various stages of the process. A detailed oxidation model of metal flames, including chemical and transport processes leading to the different species and different metal (oxide) particle sizes and shapes, can only be obtained by *in situ* measurements directly in the flame. Measured oxidation states at specific flame positions can then be correlated with recently established *in situ* nanoparticle size distributions, giving a more complete picture of the combustion stages (Hagen *et al.*, 2025 ▸). Here, synchrotron-based characterization techniques offer the opportunity to analyse, depending on the chosen method, samples with bulk as well as surface sensitivity in a time and spatially resolved manner. The high time resolution enables probing flames unstable beyond the minute time scale, while spatially resolved data can provide vital information on phase distribution in the flames.

In this study, we present a new approach to analyse the combustion process of iron by applying rapid *in situ* XAS to scan cross-sections of the flame of a laminar Bunsen-type iron dust burner. It provides information on the oxidation state and phase distribution of iron (oxide) particles at different heights and distances to the centre of the flame, representing different stages of combustion. Quick-scanning extended X-ray absorption spectroscopy (QEXAFS) enables the tracking of fast occurring changes in the flame density and selection of good spectra for further analysis due to its extremely high time resolution. The obtained results could serve as a direct input for the generation and validation of models for the combustion of iron–air mixtures. These are necessary for optimization of technical parameters for future industrial applications and cannot be obtained otherwise.

## 2. Materials and methods

### 2.1. Materials and SEM characterization

Iron powder (99.5% purity) was obtained from Metallpulver 24 (Sankt Augustin, Germany), with a mean particle size of approximately 15 µm. The powder was dried for at least one hour at 60°C to remove moisture. The initial size distribution is provided elsewhere (Fedoryk *et al.*, 2023 ▸).

Initial and combusted micrometre-sized particles were investigated using a LEO 1530 Gemini scanning electron microscope equipped with a Schottky field emitter and operated at an acceleration voltage of 5 kV to 10 kV. For this, the powder was mounted on a metal stub using a sticky carbon disc. Prepared samples were then transferred to the scanning electron microscopy (SEM) chamber for analysis under high vacuum.

### 2.2. QEXAFS characterization

The *in situ* QEXAFS spectra were recorded at the P64 beamline of the PETRA III synchrotron radiation source (DESY, Hamburg, Germany) (Caliebe *et al.*, 2019 ▸). QEXAFS was necessary in order to account for the rapid changes in the absorption spectra occurring during the experiment. Spectra were recorded in transmission mode at the Fe *K* absorption edge, in the energy range 6990–7761 eV. The energy of the X-ray photons was selected by an oscillating Si (111) channel-cut monochromator (CCM), and the beam size was approximately 1 mm (vertical) × 1 mm (horizontal). The CCM oscillation frequency was set to 5 Hz and the analog-to-digital converter (ADC) sampling frequency was set to 2 MHz. The measured X-ray absorption spectra were first sorted to remove those with unstable particle density in the beam (*e.g.* during the ignition and flameout events) and the spectra of the steady flame were averaged using *JAQ* software 3.3.49v5.3 by Oliver Müller (BU Wuppertal) (Bornmann *et al.*, 2019 ▸). Energy alignment, using the spectra of Fe foil recorded simultaneously with the sample spectra, and rebinning of the spectra were performed using *Fastosh* 1.05 (Landrot & Fonda, 2025 ▸). The rebinned spectra were then imported and normalized using the *Athena* 0.8.056 program from the *IFEFFIT* software package (Ravel & Newville, 2005 ▸). Linear combination analysis (LCA) was performed in *Athena* on the normalized spectra in the range 7110 and 7170 eV to obtain the bulk phase concentration of the different iron oxides. For this purpose, α-Fe_2_O_3_ and Fe_3_O_4_, FeO, and Fe^0^ references were measured and used as references for LCA. Measured spectra of the flame showed slight dampening of spectral features (within 10% of the maximum intensity) while relative intensities and peak positions stayed the same as in the reference spectra. The dampening was caused by an inhomogeneous sample density in the beam, and not due to chemical effects. To mitigate the dampening effect on the quantification, the Fe^0^-reference spectrum used for the LCA was extracted from the dataset measured during iron seeding but without ignition to obtain a reference with a comparable dampening degree to the experimental spectra of the flame. Due to the spectral dampening in the datasets and the combinatory nature of the oxide species, linear combination of four species led to increased misfit. Therefore, based on the thermodynamic instability of hematite (α-Fe_2_O_3_) in the expected temperature range, it was excluded from this analysis.

Since XAS in transmission mode provides line-of-sight (LOS) integrated information across the flame, the LOS molar fractions obtained from LCA were subsequently reconstructed into local radial phase distributions by inverse Abel transform assuming a fully cylindrical symmetry. The transformation was performed using a discretized chord-length formulation with Tikhonov regularization; the regularization strategy follows the inverse tomography method described by Daun *et al.* (2006 ▸). To suppress numerical noise, the reconstructed fields were smoothed using a Gaussian filter and interpolated onto a refined spatial grid for visualization. Reconstruction consistency was verified by forward projection of the reconstructed radial fields, yielding negligible deviations from the measured LOS-integrated data.

### 2.3. Bunsen burner used for the synchrotron experiment

In this study, a laboratory-based Bunsen-type iron dust burner, originally introduced by Fedoryk *et al.* (Fedoryk *et al.*, 2023 ▸; Braig & Fedoryk, 2023 ▸), was modified and employed to stabilize a self-sustained stoichiometric laminar iron dust flame at the synchrotron (Braig & Fedoryk, 2023 ▸). The stoichiometry of the iron dust flames was defined with respect to complete oxidation to hematite (α-Fe_2_O_3_), *i.e.* to an equivalence ratio and/or an air-to-fuel ratio of unity. Iron powder was stored in a cylindrical tube with a diameter of 16.4 mm and pneumatically dispersed by an air-knife seeder adapted from Goroshin *et al.* (1996 ▸). A piston, driven by a stepper motor via a threaded shaft, continuously fed the powder upwards into the dispersion zone. At this stage, the oxidiser gas, *i.e.* synthetic air, was accelerated through a circular slit of 30 µm width, generating high velocities that suspended the powder in a homogeneous aerosol. Downstream, a 45° constriction guided the aerosol into a pilot tube with an inner diameter of 5.6 mm and a length of 10 cm, before it entered the combustion tube of 20.5 mm inner diameter and 35 cm length. This two-stage tube system reduced wall deposition and ensured that detached agglomerates were collected separately. The outlet of the combustion tube was surrounded by a coflow tube with a diameter of 58.8 mm, supplying an air coflow that stabilized the flame at the burner rim and shielded it from external perturbations. Both oxidiser and coflow were supplied at ambient temperature, with air at atmospheric composition, *i.e.* 21% O_2_ and 79% N_2_, as the oxidiser. The mean exit velocity at the burner outlet was set to 40 cm s^−1^, and the co-flow was operated at the same velocity to maintain symmetry. Ignition was achieved by a small, non-premixed CH_4_/air pilot flame, which was switched off once the iron flame became self-sustained. Burned particles were collected downstream in a dedicated ATEX-certified extraction unit. For safety and optical access, the entire burner was placed in a transparent polycarbonate enclosure with 50 µm-thick polyimide windows for X-rays. This configuration enabled long-term stable operation of stoichiometric iron dust flames under well defined and reproducible boundary conditions suitable for synchrotron-based diagnostics.

## 3. Results and discussion

### 3.1. Burner setup and preparation

**Figure 1:**
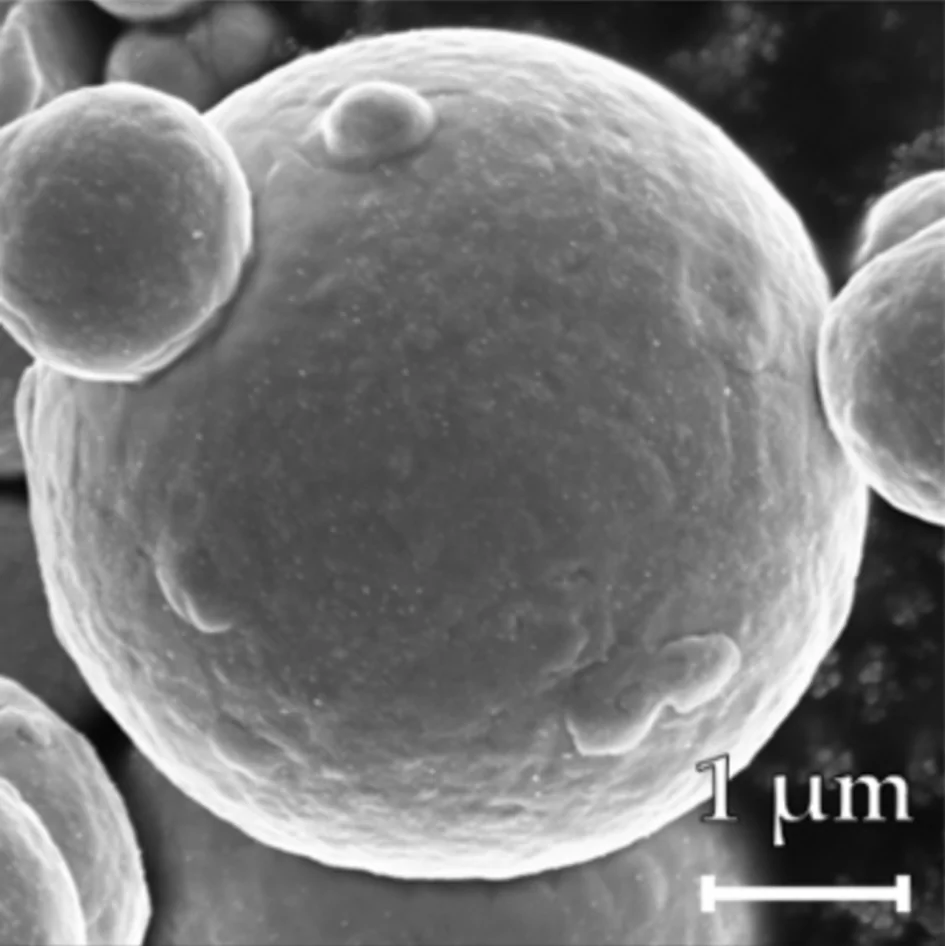
Representative SEM image of an iron powder particle before burning.

For the combustion experiments, spherical metallic iron particles, as shown in the SEM image in Fig. 1 ▸, were used. The number-based mean particle diameter (15 µm) with a size distribution determined by shadowgraphy are given by Fedoryk *et al.* (2023 ▸). SEM imaging (Fig. 7) of the collected particles showed intact particles with iron oxide nanoparticles attached to the surface of the main particle.

**Figure 2:**
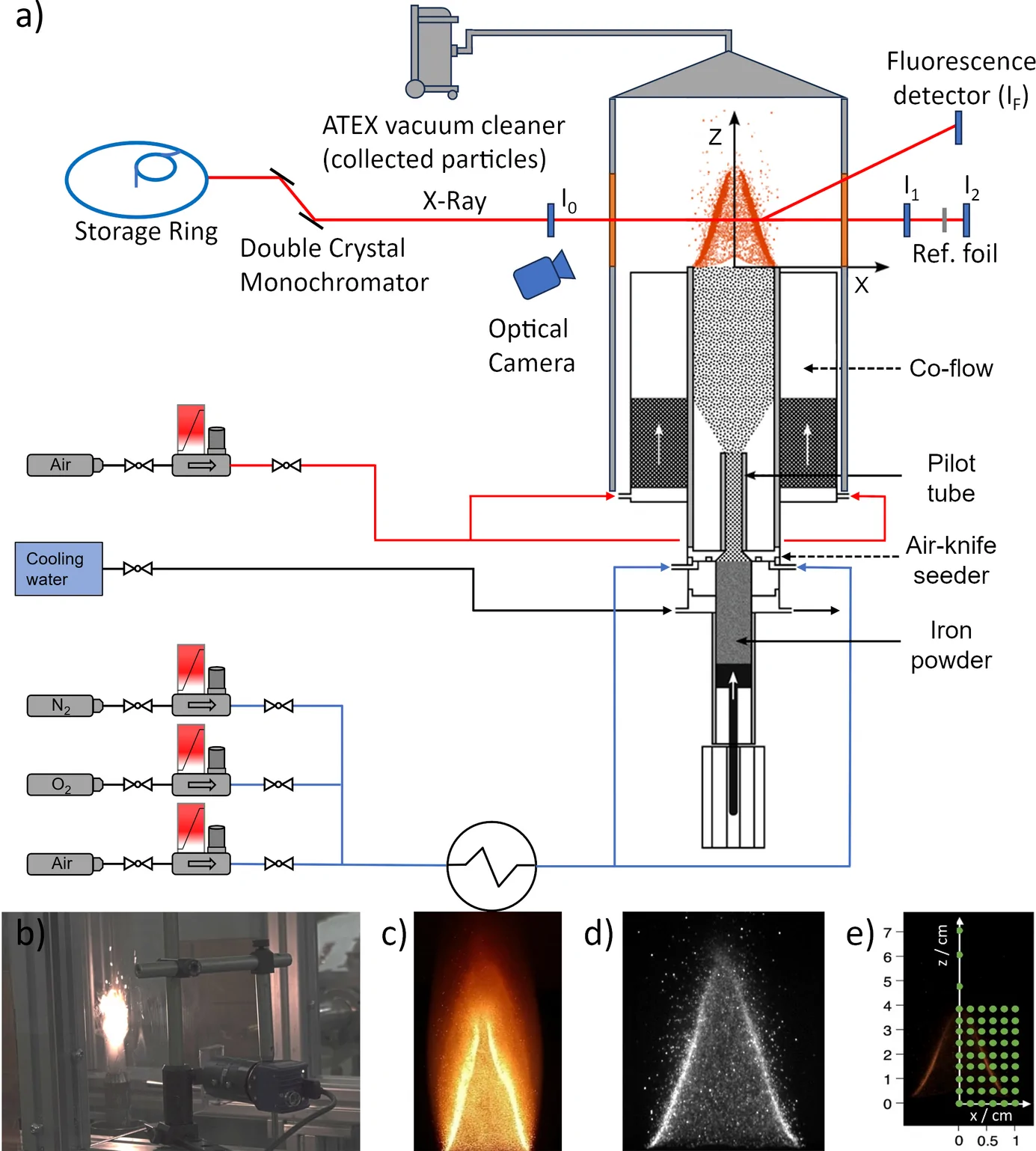
The *in situ* synchrotron experiments. (*a*) Basic schematic of the experimental setup including X-ray components, mass flow controllers, optical camera and the seeding system. Experiment under stochiometric conditions with 21% O_2_ and 79% N_2_ as oxidizer. (*b*) Photograph of the ignition (with a faint pilot flame to the bottom-right) and the 10 Hz optical camera setup. (*c*) Single-shot flame image during the measurement for visualizing flame shape. (*d*) Monochrome single shot image for particle tracking. (*e*) Measurement grid overlapping a single-shot flame image.

The modified Bunsen burner setup allows synchrotron-based non-invasive *in situ* characterization by X-rays while keeping representative combustion conditions that are applicable in industry (Fig. 2 ▸). A basic schematic of our concept is shown in Fig. 2 ▸(*a*), illustrating the principle of incorporating X-ray-based probes into the burner while adhering to the strict safety and cleanliness requirements of synchrotron-based research facilities.

More details of the experimental setup, including pictures, as well as a short film of the ignition process and a stable flame, can be found in the supporting information. The installed mass flow controllers allow dosing four different gases: pressurized air, nitro­gen (N_2_), oxygen (O_2_) and methane (CH_4_). Combustion in this setup is designed to work without any additional preheating or permanent pilot flame. Methane as the pilot flame fuel has been integrated for the initial ignition of the metal fuel based flame and is turned off directly after stabilization of the metal flame—a concept allowing reliable remote ignition for operation in X-ray controlled areas. Pressurized air provides co-flow surrounding the outlet tube to increase the stability of the flame and shield it from external disturbances during the measurement. Separate dosing of oxygen and nitro­gen allows easy variation of the fuel-to-oxidiser ratio without influencing the flow regime. In order to collect the burned particles for further analysis and protect the surrounding equipment, the burner area was enclosed from the sides and at the top in a polycarbonate (PC, Makrolon) enclosure with X-ray transparent polyimide windows for incident and transmitted as well as fluorescence (at 90° to the incident beam) photons. The enclosure was connected at the top to an ATEX (ATmosphères EXplosives) compliant vacuum cleaner to collect the burned particles. At the same time, it allowed unobstructed air flow from the bottom in order not to disturb the flame, as seen in Appendix *A*[App appa] (Fig. 6). Apart from health protection reasons, this was necessary due to the highly sensitive equipment at the typical synchrotron beamline, which could be damaged by metal particle dust. The major technical complication for the synchrotron measurements stems from the dimensions of the burner setup, which cannot be mounted on an optical table as is usually done for the majority of synchrotron experiments. This severely limits the choice of the potential beamlines for such measurements. In our case, the detectors for the XAS measurement were mounted on a robotic arm, placed behind the burner or directly outside the enclosure, according to Fig. 6, to allow for transmission as well as fluorescence measurements. An ionization chamber I_0_ and a tube connected to a fore vacuum pump (so called ‘flight tube’ to reduce absorption of X-rays by air) were mounted on the optical table. The reference iron foil spectrum for energy alignment was measured in transmission mode with a Passivated Implanted Planar Silicon (PIPS) diode mounted after the second ionization chamber on the same plate held by a robotic arm installed behind the optical table of the beamline and the burner setup. For the later correlation of the obtained spectra to the flame cross section an optical camera was installed along the visual axis. The complete burner assembly was mounted on a remotely controlled *XZ*-motorized linear stage to allow precise positioning of the flame relative to the fixed X-ray beam.

### 3.2. Mapping a stoichiometric flame by QEXAFS

**Figure 3:**
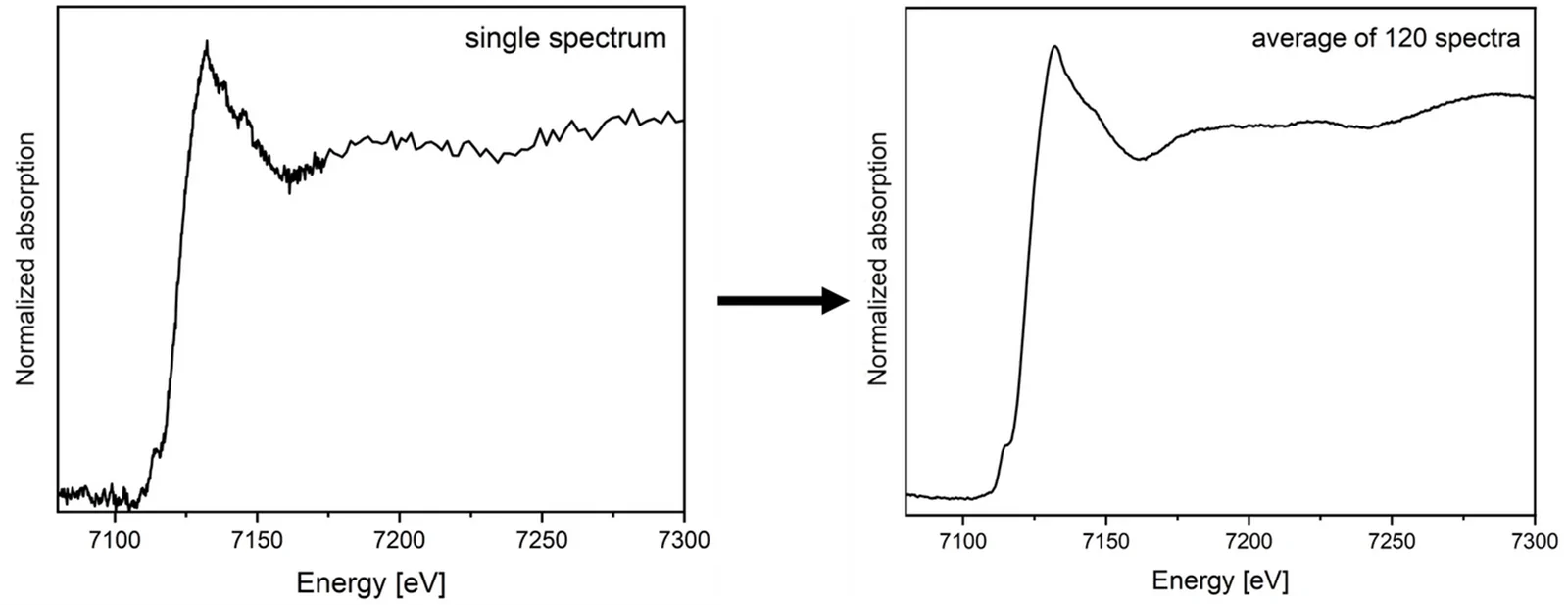
Comparison of the raw, non-averaged spectrum and the spectrum after averaging all valid spectra at one position (*Z* = 2.0 cm and *X* = 0.6 cm).

For accurate mapping of oxidation states of such a volatile and fast changing system as an iron dust flame we used QEXAFS as a technique with short measurement times (Bornmann *et al.*, 2019 ▸; Schroer *et al.*, 2003 ▸). An optimal ratio between spectra collection speed and data quality was found at 5 Hz oscillation frequency of the CCM with data collection in transmission mode at 2 MHz sampling rate, using ionization chambers I_0_ and I_1_, as well as the reference PIPS diode I_2_. The acquisition sequence was started before ignition of the flame and stopped after flameout, and all spectra of the stable flame taken between these events were averaged together for improved data quality, see Fig. 3 ▸. Spectra of unstable flame, typically measured during ignition or flameout events, were identified as deviating from typical XAS spectral shapes based on a sigmoid function. The occurrence of these spectra emphasizes the need for high measurement speeds, as they could compromise the reliability and accuracy of the results substantially.

A 10 Hz camera, as seen in Fig. 2 ▸(*b*), was used to visualize the flame and determine its position and dimensions. Furthermore, the camera allowed tracking of any major movements [Fig. 2 ▸(*c*)], flashbacks or flameouts, which could potentially impact the results when included in the averaged datasets and evaluation procedure. In order to identify the dimensions of the flame and to establish a proper measurement pattern, a single flame image was taken as shown in Fig. 2 ▸(*d*), as well as an X-ray absorption scan across the burner nozzle. For the visible flame, a diameter at the bottom of 2.0 cm, corresponding to the size of the outlet nozzle, was determined and a height of around 4.0 cm was estimated. From this, a grid of 57 points, shown in Fig. 2 ▸(*e*), was determined sufficient for measuring the stoichiometric flame, covering the entire flame region and including multiple points outside the flame cone to track the downstream evolution of iron species.

X-ray absorption near-edge structure (XANES) spectra were collected at the aforementioned points of the flame grid, followed by normalization and LCA, as described in the *Methods* section[Sec sec2]. In the following only three reference phases were used for the fitting, namely Fe^0^, FeO and Fe_3_O_4_, as precise deconvolution of the four-component system including hematite (α-Fe_2_O_3_) is not robust and leads to large errors as seen in Fig. 8. In principle, XAS can distinguish between magnetite (Fe_3_O_4_) and wüstite (FeO)/hematite (α-Fe_2_O_3_) in non-distorted high quality datasets. However, in terms of spectral shapes and especially the rising edge position which significantly contributes to the LCA, the XANES spectrum of Fe_3_O_4_ resembles a combination of α-Fe_2_O_3_ and FeO, given that it contains both Fe^2+^ and Fe^3+^ species. In this work α-Fe_2_O_3_ was not included in the fitting process as it is not expected to form under the conditions the particles are exposed to in our experiment. At the high temperatures, which can be assumed from simulations for the same flame conditions as used in this work (Hazenberg *et al.*, 2026 ▸) to be above 1220°C, α-Fe_2_O_3_ would decompose and is therefore unstable. Moreover, according to the phase diagram, the maximum molar oxygen-to-iron ratio in the investigated flame remains below the stability limit of both hematite and maghemite (γ-Fe_2_O_3_) (Hazenberg *et al.*, 2026 ▸; Tang *et al.*, 2011 ▸; Ketteler *et al.*, 2001 ▸; Von Bogdandy & Engell, 1971 ▸).

Extended X-ray absorption fine structure (EXAFS) data could also be extracted and Fourier transformed (FT) to obtain pseudo radial distribution functions (Fig. 9). Due to the high sensitivity of EXAFS to temperature and dampening of the spectra due to flame inhomogeneity, the evaluation is limited to qualitatively comparing the FT EXAFS with reference data, with the trend confirming the XANES analysis results.

**Figure 4:**
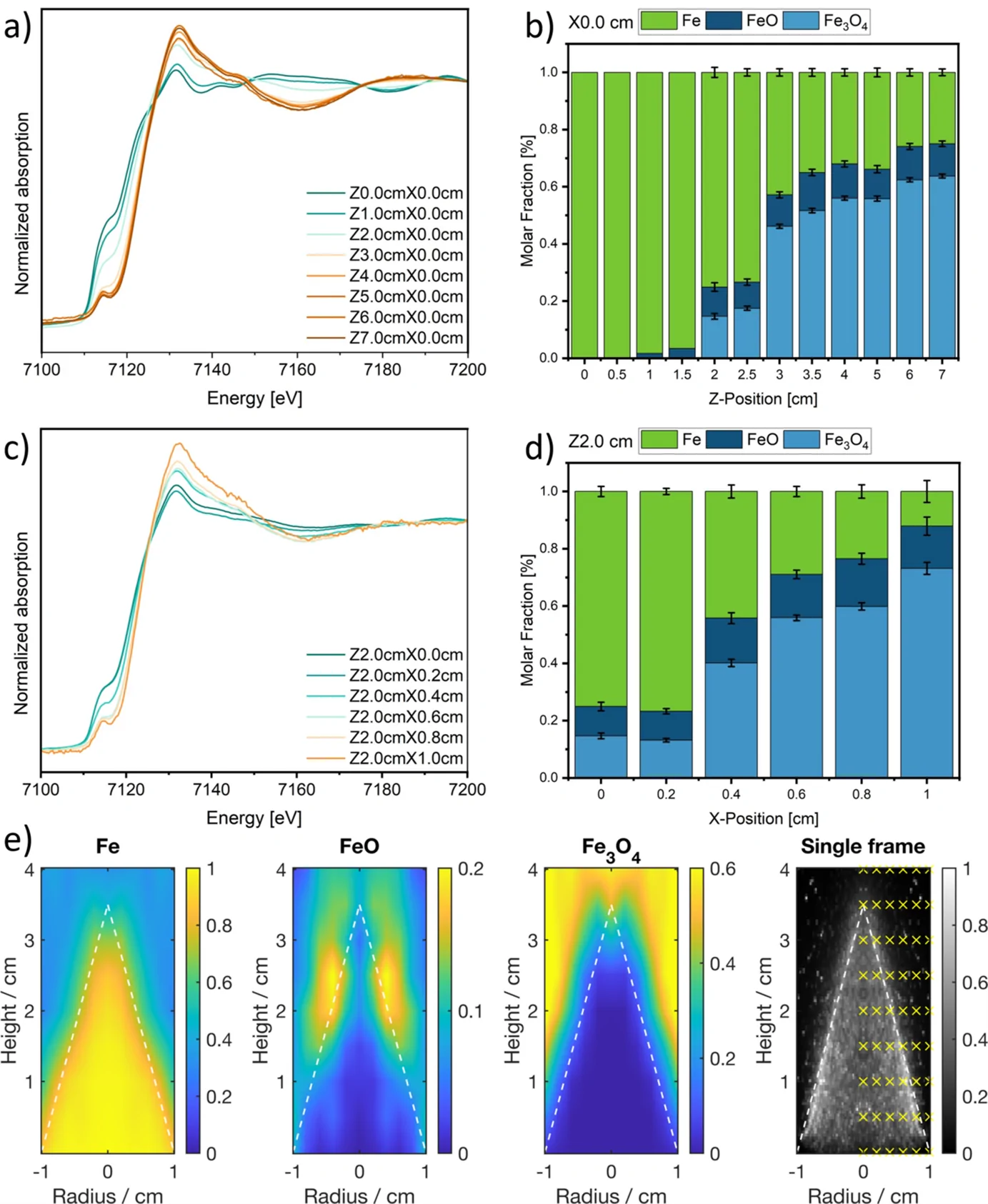
(*a*) Normalized XAS spectra at constant horizontal position *X* = 0.0 cm. (*b*) LCA of XAS spectra at *X* = 0.0 cm. (*c*) Normalized XAS spectra at constant vertical position *Z* = 2.0 cm. (*d*) LCA of XAS spectra at *Z* = 2.0 cm (error bars do not account for the spectral dampening). (*e*) Abel transform of the complete flame. Note the different scales for Fe, FeO and Fe_3_O_4_.

When discussing XAS mapping results, first the phase composition and Fe oxidation state changes along horizontal and vertical axes of the flame were evaluated. While vertically moving up along the flame axis (*X*-position at 0.0 cm) from *Z* = 0.0 cm to *Z* = 7.0 cm (only the central flame axis at *X* = 0 cm was probed above *Z* = 4 cm), we could observe a gradual change in spectra from metallic iron (Fe^0^) to oxidic iron, seen in the shifts of the rising edge and the white line (the first, most intense peak after the rising edge), as well as the white line intensity decay [Fig. 4 ▸(*a*)]. According to the quantification via LCA [Fig. 4 ▸(*b*)], at the very bottom of the flame only metallic iron (Fe^0^) could be found, which was gradually transformed to its oxidized counterparts when moving upwards. Formation of FeO occurred as the first stage of the combustion process soon after and only when the particles are transported further outwards an oxidation to Fe_3_O_4_ was observed. A strong and rapid increase in oxidation state was detected when reaching *Z* = 3.0 cm, which leads to the conclusion that the beginning of the reaction zone was located between *Z* = 3.0 cm and *Z* = 3.5 cm, the latter coinciding with the top of the visible flame cone. After leaving the reaction zone, mainly an increase in the Fe_3_O_4_ fraction was found, while the FeO fraction remained mostly constant, which might be due to the ongoing formation of FeO and its subsequent conversion towards Fe_3_O_4_. Looking at the total conversion, a maximum amount of approximately 64% ± 2% Fe_3_O_4_ and 11% ± 2% FeO was detected.

The horizontal variation of iron oxidation state and phase composition is discussed taking the spectra recorded at *Z* = 2.0 cm as an example. Similarly to the vertical gradient, we could spot a visible change in spectral features from metallic iron to iron oxide while moving to the outer regions of the flame [Fig. 4 ▸(*c*)]. LCA [Fig. 4 ▸(*d*)] showed the most prominent increase in oxidation state between *X* = 0.4 cm and 0.6 cm, which indicates particles entering the reaction zone (visible flame border at *X* approximately 0.45 cm). The oxidation was therefore incomplete, with about 10–25% Fe^0^ left depending on the probed position (*Z* > 2.0 cm and *X* = 1.0 cm). A limitation of the line-of-sight XAS measurement concept is highlighted by the iron oxide found in the spectra corresponding to the core of the flame, *X* = 0.0 cm to *X* = 0.2 cm, where no oxidation is expected to take place. The origin of this anomaly is the averaging nature of transmission XAS with the beam crossing the reaction zone twice, including the front and the back flame boundaries. To resolve the LOS problem and allow visualization of cross-cuts, an inverse Abel transform was applied to the LOS projection of Fe states [Fig. 4 ▸(*e*)]. The transformation assumes an approximately axisymmetric, time-averaged flame structure, which is supported by previous broadband imaging of comparable laminar iron dust flames of Bunsen-type (Hagen *et al.*, 2025 ▸). The resulting cross-sectional image displays the deconvoluted fractions of each species across the flame.

In the left-most image of Fig. 4 ▸(*e*) the Fe^0^ fraction is shown and, as expected for the line-of-sight corrected flame cross-section, no oxides are visible in the core regions of the flame. With respect to the oxides, FeO is found on the border of the visible flame zone, with the maximum concentration at about *Z* = 1.5 cm to *Z* = 3 cm. Qualitatively, the FeO concentration peaks at the reaction front/zone and decreases further outward as FeO is further oxidized to magnetite (Fe_3_O_4_. This is in line with Fe_3_O_4_ appearing roughly at the visible flame border, very close to the FeO peak zone and the visible flame front. The most pronounced FeO–Fe_3_O_4_ gradient appears to be located approximately 0.5 cm outside the visible flame border [Fig. 4 ▸(*e*)], with oxidation process continuing well outside the visible flame zone height of 4 cm [Fig. 4 ▸(*b*)]. This downstream oxidation process is to be expected, since the stoichiometric flame conditions refer to the highest oxidation state of iron as α-Fe_2_O_3_, leaving sufficient oxygen in the hot post-flame gases to further oxidize FeO to Fe_3_O_4_.

**Figure 5:**
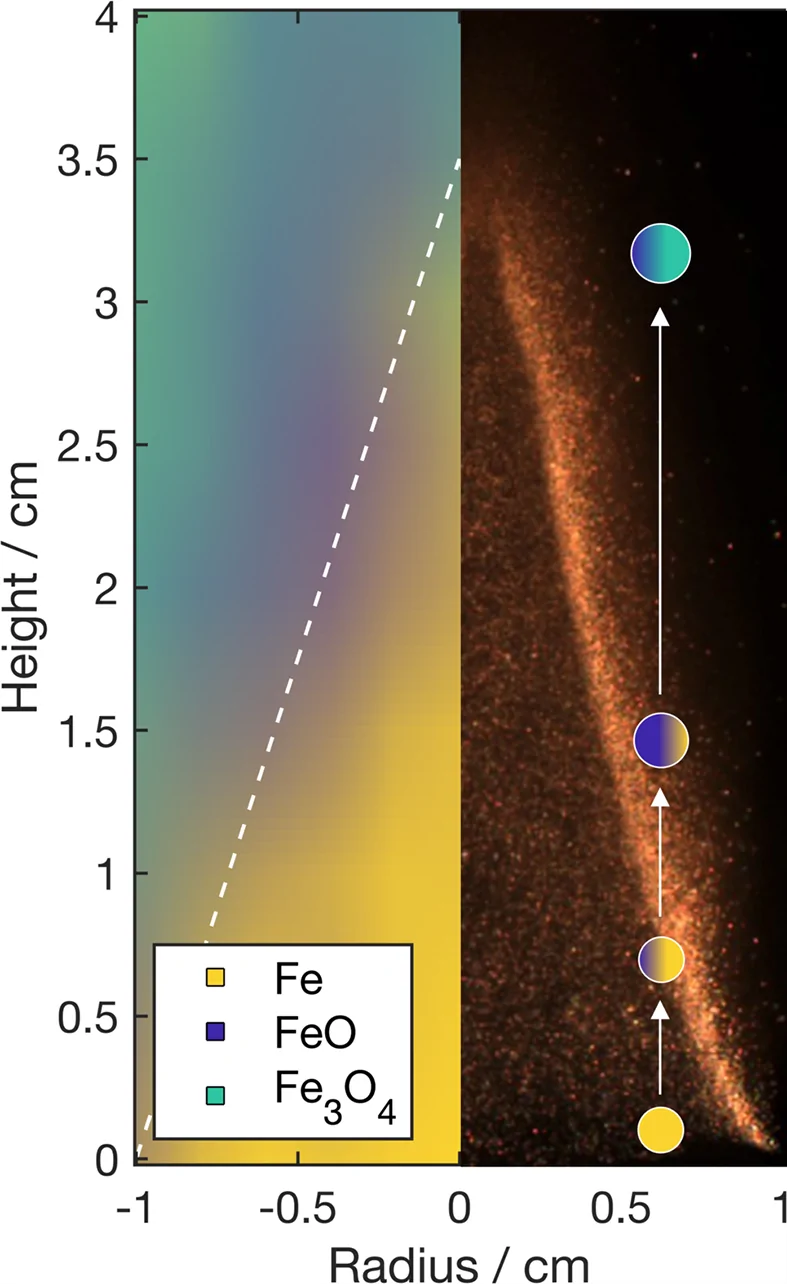
Average phase composition along the flame cross-section and the corresponding single-shot image.

**Figure 6:**
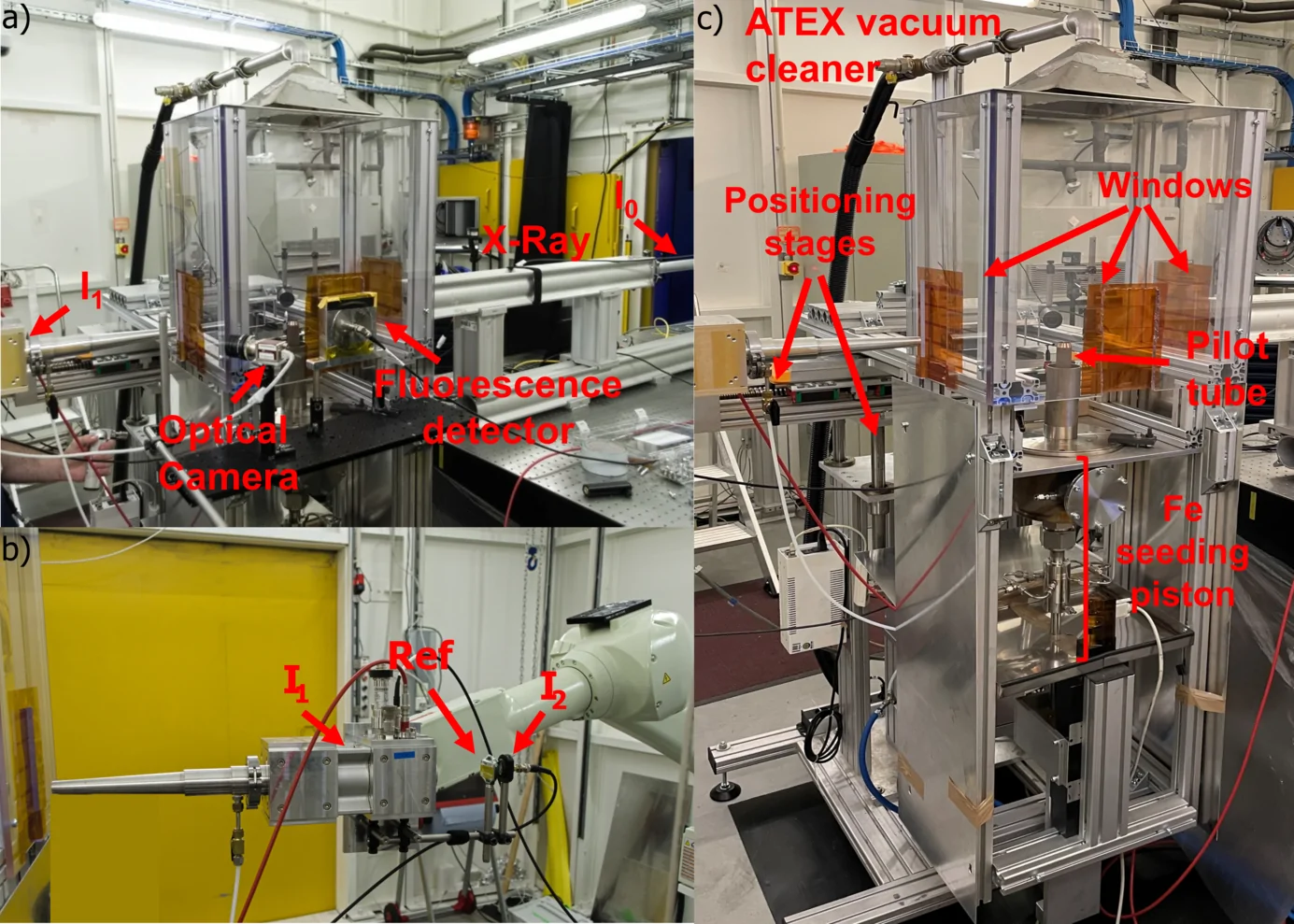
(*a*) Position of the setup relative to the optical table of the beamline. (*b*) Detector placement and position of the reference foil. (*c*) Complete setup at the P64 beamline at DESY (Hamburg).

**Figure 7:**
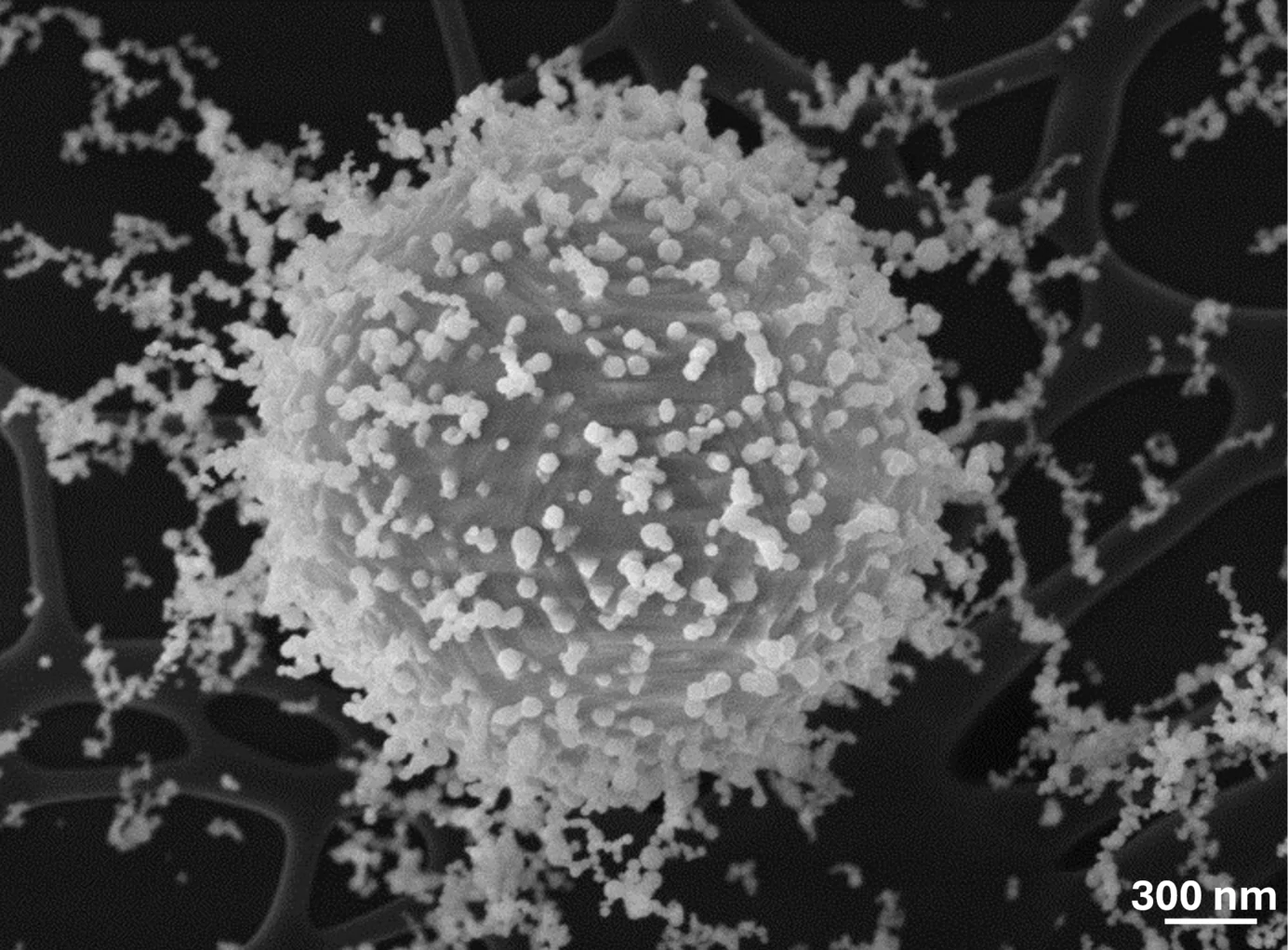
A representative SEM image of an iron powder particle after the burning process.

**Figure 8:**
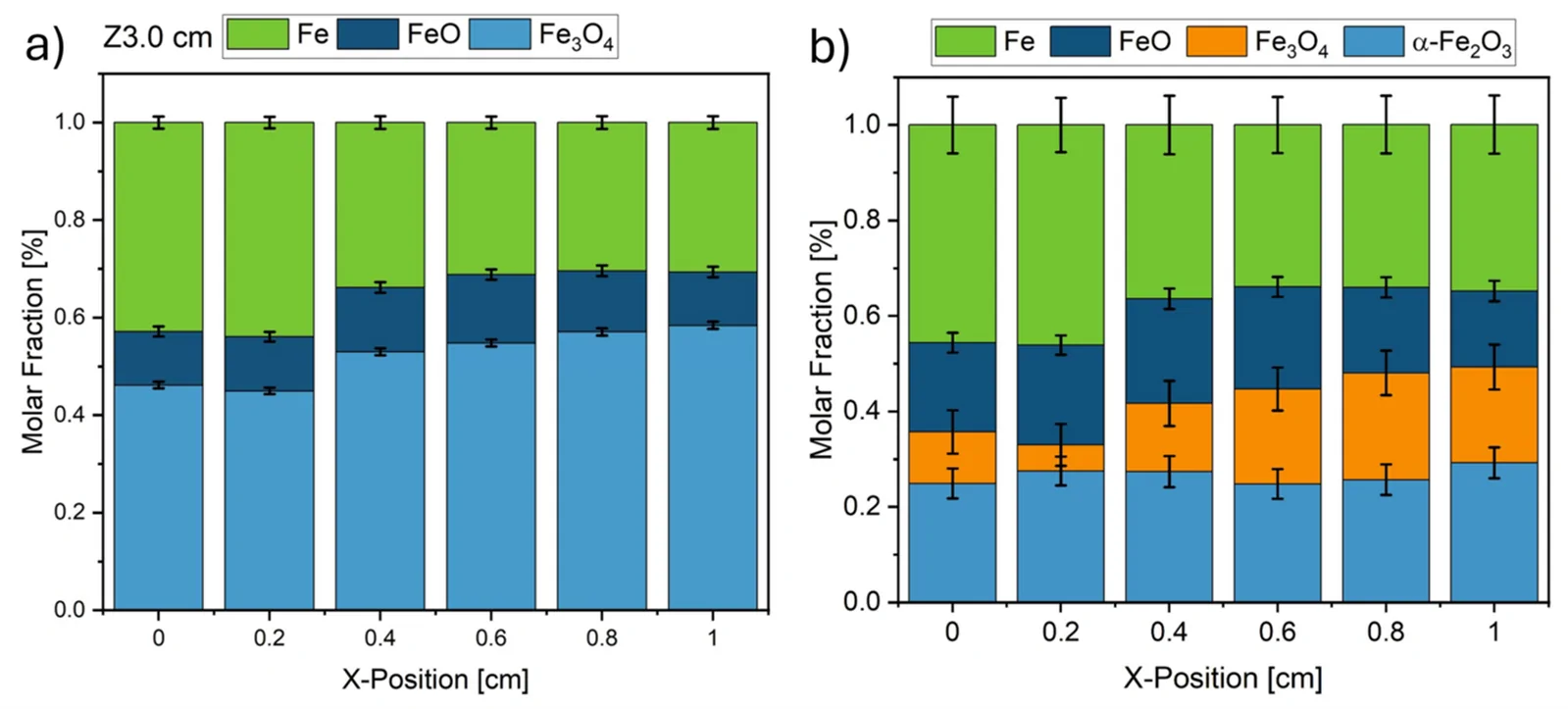
(*a*) Flame composition profile at a height (*Z*) = 3.0 cm above the burner nozzle when evaluated using LCA with three references. (*b*) LCA results at the same position including additionally α-Fe_2_O_3_ as reference. Error bars do not account for the spectral dampening.

**Figure 9:**
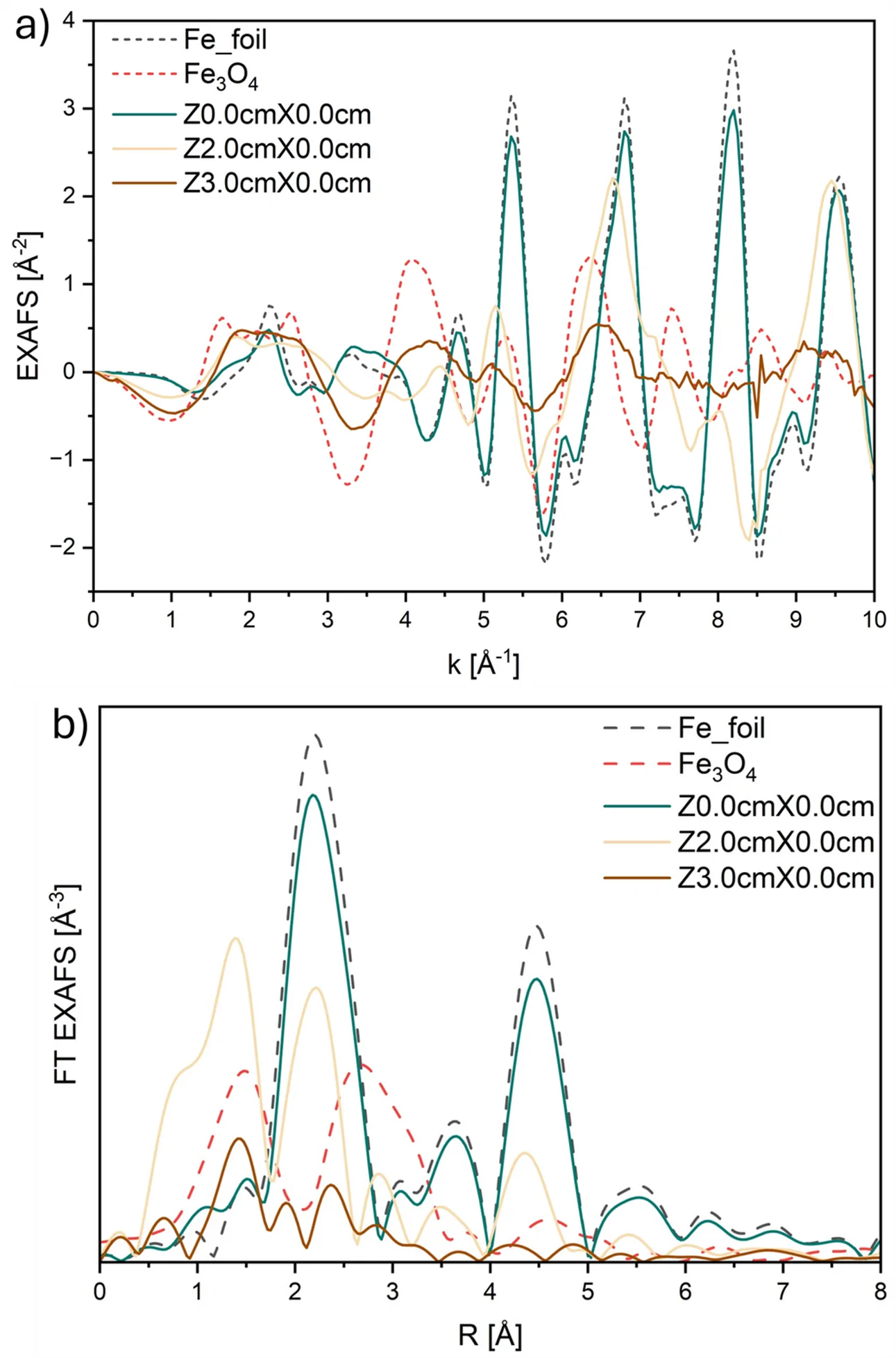
(*a*) *k*^2^-weighted EXAFS functions and (*b*) Fourier transformed (FT) EXAFS data of selected spectra measured along the flame axis and metallic iron (Fe^0^) and magnetite (Fe_3_O_4_) references. *k*-range from 2.5 to 10.

In a next step, as shown in Fig. 5 ▸, the fractions of the three individual phases were combined again to create an image representing the average oxidation state along the visual cross-section. In addition, the occurring processes are also schematically depicted in a single-shot image of the measured flame to provide a more intuitive overview of the spatial evolution. We can see that the transition from metallic iron (Fe^0^) to wüstite (FeO), according to equation (1), dominates the region around the flame border. Once the particles had left the flame front the oxidation of the FeO phase towards magnetite (Fe_3_O_4_) [equation (2)] dominated, while the previous reaction continued to a lesser extent,
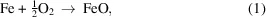

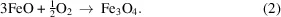
Further oxidation to hematite α-Fe_2_O_3_ did not occur in this region due to the aforementioned stability limitations and is expected to occur downstream as a final oxidation step at lower temperature. The continuation of the oxidation was observed by different groups by *ex situ* characterization of sampled particles. They showed the formation of core-shell structures for the particles with a metallic core and a shell of a mixed composition of the different oxides. Phase analysis showed further decrease of the wüstite and metallic iron fraction compared with the outermost point shown in this work and a corresponding increase for hematite (Sperling *et al.*, 2025 ▸; Deutschmann *et al.*, 2024 ▸; Buchheiser *et al.*, 2023 ▸).

## 4. Conclusion

In this study, we have presented an approach enabling *in situ* characterization of iron oxidation states and corresponding phases by synchrotron X-ray absorption spectroscopy in an iron dust flame in a Bunsen-type burner, together with optical camera images, which was made possible by the development of a dedicated setup. We were able to map iron oxide phases in a stoichiometric flame. This lays the ground for the extension to other metal flames, which are notoriously difficult to characterize *in situ*. The combination of *in situ* quick-scanning XAS, advanced analysis, optical camera and a tailor-made iron powder burner allowed experimentally spatially resolved insights into phase transformations of iron (oxides) in the laminar Bunsen-type flame across the flame cross-sections. For the first time, the gradients in oxidation of metallic iron (Fe^0^) and formation of wüstite (FeO) and magnetite (Fe_3_O_4_) could be non-invasively visualized directly in the flame, at relevant temperatures, pressures and oxygen concentrations.

We detected distinct changes in the oxidation state and phase composition, depending on the probed position in the flame. Applying an inverse Abel transform resolved the line-of-sight problem, enabling maps of the Fe flame cross-sections to be visualized. After the transformation, the slice of the flame showed a distinct core with Fe^0^, the reaction zone with a visible flame front and maxima in FeO concentration, and outwards progression of FeO gradual oxidation to Fe_3_O_4_. A strong increase in oxidation degree (to FeO) could be observed when the particles entered the reaction zone (coinciding with the maximum luminosity region), and the oxidation continued when moving outwards, while the centre remained predominantly metallic. Further oxidation, mainly conversion to Fe_3_O_4_ with a steeper concentration gradient, was detected after the particles left the visible flame zone. In the reaction zone, the FeO fraction peaks and then decreases downstream, consistent with its further oxidation to Fe_3_O_4_. Hence, the obtained data for the first time allows one to relate predicted and experimental flame parameters, such as temperature and oxygen amount, to the actual spatial distribution of iron (oxide) phases directly in the flame volume. This can now be employed to optimize flame properties and potentially decrease nanoparticle formation, thereby improving the efficiency of the oxidation process.

This work lays the basis for further non-invasive investigations of metal flames with X-ray techniques in general. Future modifications to the setup are expected to allow the usage of additional, complementary characterization techniques, such as *in situ*X-ray diffraction or small-angle X-ray scattering, which would additionally give us the possibility to more precisely distinguish between Fe^2+^- and Fe^3+^-containing amorphous/crystalline phases with similar spectral features. Next, it can also be adapted for different oxidiser compositions or metals and, when using X-ray scattering techniques (*e.g.* XRD, SAXS), to the combustion of other solid fuels such as aluminium and silicon, which are promising for a diversified feedstock for retrofitted coal power plants. Further, metal powders are used in many applications where rapid oxidation or burning processes occur, apart from energy storage, such as in rocket propellants, fuel additives or explosives, analysis of which is so far only done either *ex situ* by the aforementioned methods or using imaging or laser diagnostics (Babuk *et al.*, 2001 ▸; Gao *et al.*, 2017 ▸; Corcoran *et al.*, 2013 ▸; Heng *et al.*, 2025 ▸; Pang *et al.*, 2021 ▸). The developed procedure and the setup could also be used to gain insights into the mechanism of oxide formation during flame spray pyrolysis. Our approach attempts to bridge this gap in characterization and establish a reliable *in situ* method for analysis of metal (precursor) combustion.

## Supplementary Material

Video of the metal burner ignition. DOI: 10.1107/S1600577526005904/ok5161sup1.mp4

Reference spectra, Illustration of spectral dampening due to inhomogeneous sample, further LCA results. DOI: 10.1107/S1600577526005904/ok5161sup2.pdf

## Data Availability

XAS data were generated at the Deutsches Elektronen Synchrotron DESY (Germany). The collected reduced (averaged and rebinned) data acquired included in this study are stored in KITopen, the central repository of the Karlsruhe Institute of Technology, and are freely available https://doi.org/10.35097/71ekatz6whesv6k6.

## Appendix A Setup used at the synchrotron

The X-ray beam, after the slit system and ionization chamber I_0_, was directed to the experimental setup using a ‘flight tube’ (rough vacuum to avoid attenuation and scattering of the beam by the atmosphere). The incident beam intensity was recorded using ionization chamber I_0_ placed on the optical table of the beamline. After passing through the sample the transmitted remaining photons were detected by ionization chamber I_1_. A PIPS diode serves as the third detector I_2_ recording the signal after absorption of the downstream placed reference iron foil, which is used as reference for energy scale alignment and calibration. Fluorescence signal from the flame was additionally collected by a PIPS detector positioned at a 90° angle relative to the incident beam; however, the transmission signal was exclusively used for data evaluation due to better quality. An optical camera was installed to visualize the flame and determine its position and spatial dimensions. A complete housing with X-ray transparent polyimide (Kapton) windows was built to protect the highly sensitive equipment present at beamlines, while an ATEX-compliant vacuum cleaner was used to collect the particles generated during the experiment. The burner was mounted on a motorized *XY* translation stage allowing the burner to be precisely positioned in the beam path. The burner itself contains a pilot tube to reduce fluctuations caused by powder falling back and then being reseeded. The Fe powder seeding system consists of a cylindrical tube with a piston that continuously pushes iron powder upwards, where it is ignited by the pilot flame (fueled by methane). Fig. 6 ▸ shows the setup used at the synchrotron.

Fig. 7 ▸ shows an SEM image of the burned iron powder (see Section 3.1). Fig. 8 ▸ shows exemplary flame composition profiles obtained by LCA of the line-of-sight spectra before applying the inverse Abel transform, while Fig. 9 ▸ shows *k*^2^-weighted EXAFS functions and Fourier-transformed EXAFS data of selected spectra (see also Section 3.2).
