# Supplementary material for: In situ mapping of iron oxidation in laminar metal fuel flames using X-ray absorption spectroscopy
Source: J Synchrotron Radiat. 2026 Jul 15;33(Pt 5):1418–27. doi: 10.1107/S1600577526005904 (PMC13549353; doi:10.1107/S1600577526005904)
Supplement: Supplementary file 1 [file s-33-01418-sup2.pdf]

JOURNAL OF SYNCHROTRON RADIATION

Volume 33 (2026)

Supporting information for article:

In situ mapping of iron oxidation in laminar metal fuel flames using X-ray absorption spectroscopy

Lukas Braun, Fabian P. Hagen, Michal Fedoryk, Malte Seitz, Björn Stelzner, Dimosthenis Trimis, Jan-Dierk Grunwaldt and Dmitry E. Doronkin

Supporting Information

In situ mapping of iron oxidation in laminar metal fuel flames using X-ray absorption spectroscopy

Lukas Braun[a, c], Fabian P. Hagen*[b], Michal Fedoryk[b], Malte Seitz[b], Björn Stelzner[b], Dimosthenis Trimis[b], Jan-Dierk Grunwaldt[a, c], Dmitry E. Doronkin*[a, c]

[a] Karlsruhe Institute of Technology, Institute for Chemical Technology and Polymer Chemistry (ITCP), Engesserstr. 20, 76131 Karlsruhe, Germany
[b] Karlsruhe Institute of Technology, Engler-Bunte-Institute, Combustion Technology, Engler-Bunte-Ring 7, 76131 Karlsruhe, Germany
[c] Karlsruhe Institute of Technology, Institute of Catalysis Research and Technology (IKFT), Hermann-von-Helmholtz-Platz 1, 76344 Eggenstein-Leopoldshafen, Germany

Correspondence e-mail: dmitry.doronkin@kit.edu, fabian.hagen@kit.edu

S1. Reference spectra and further LCA

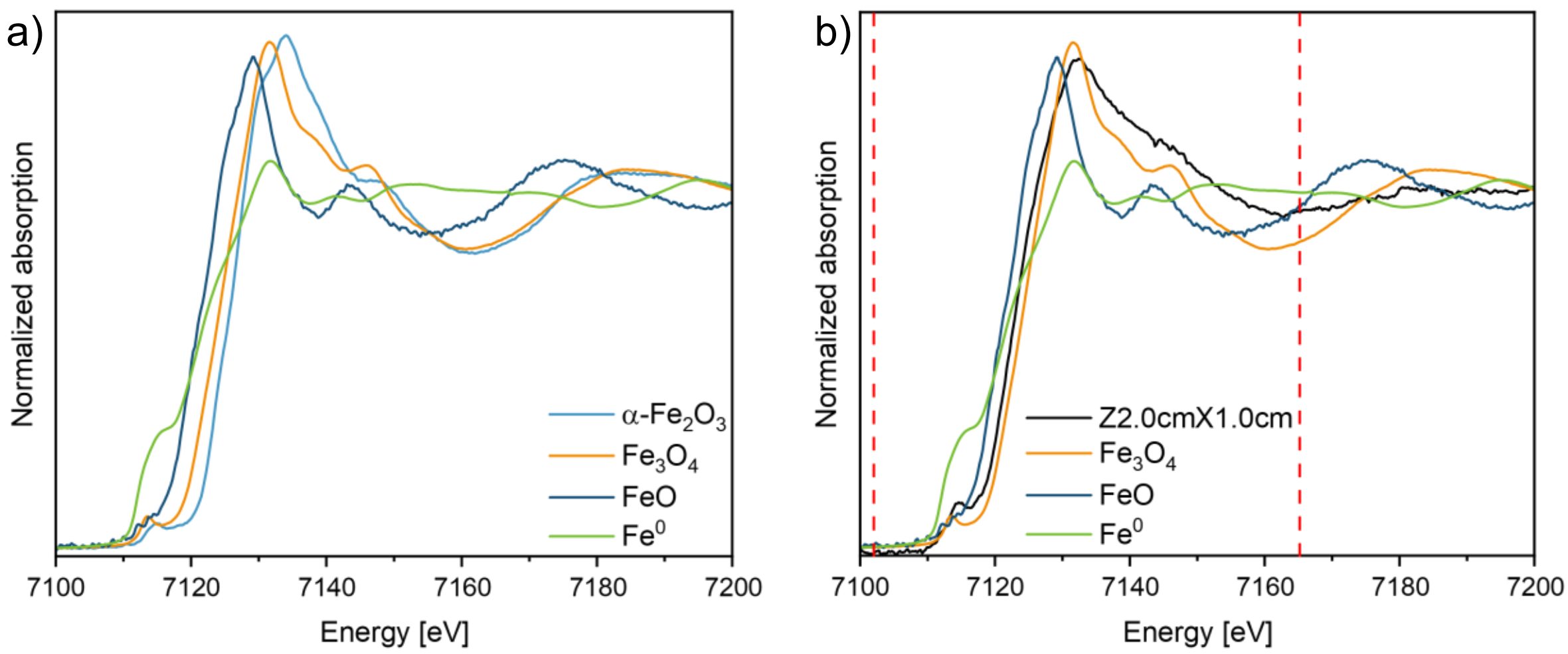

Figure S1 a) Reference spectra of α-Fe_2O_3, Fe_3O_4, FeO and Fe^0 measured at the P64 beamline, b) Exemplary spectrum of the flame with relevant reference spectra and the fitting range for linear combination analysis (LCA) marked by red vertical line.

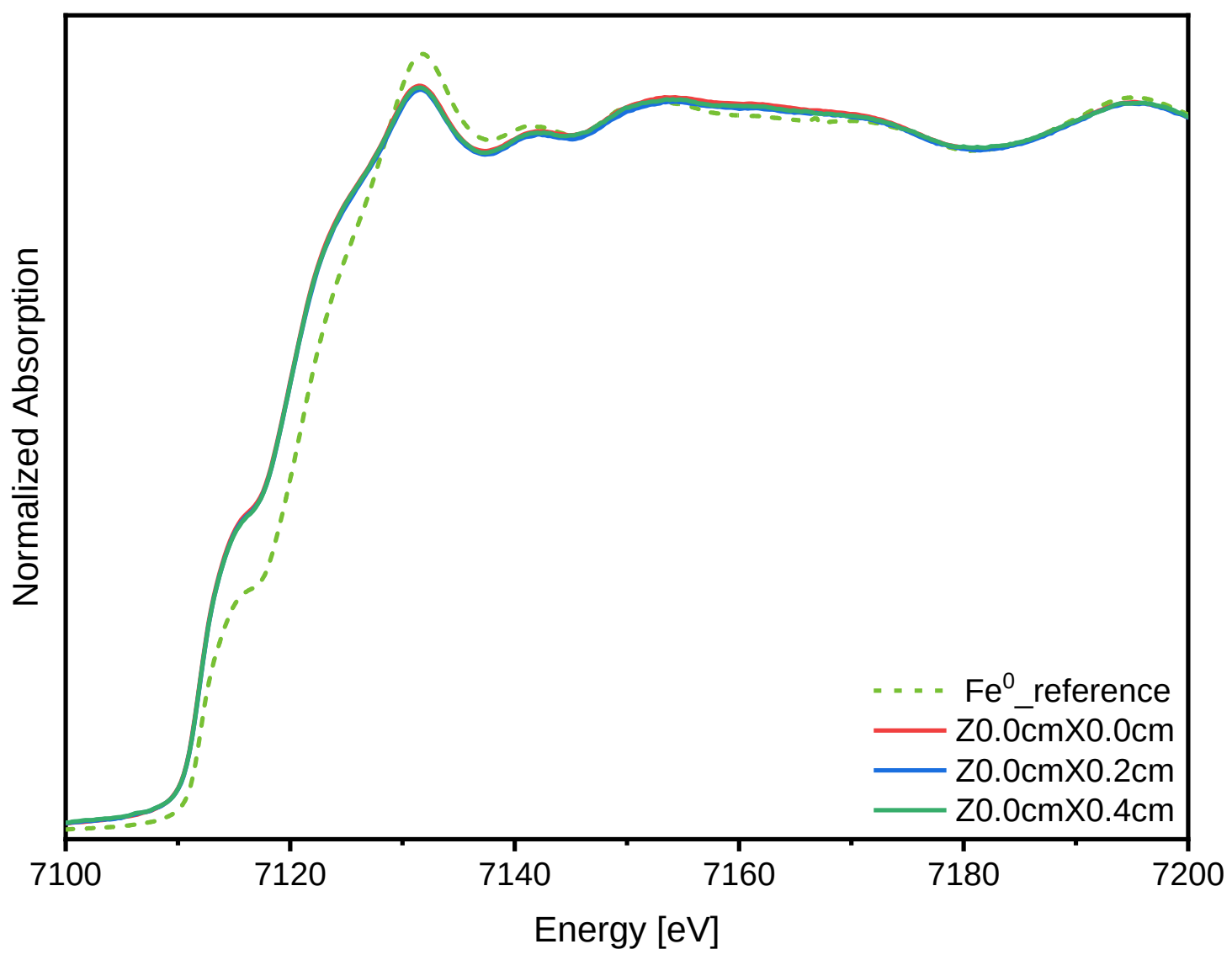

Figure S2 Comparison of the metal foil spectra (Fe^0 reference, dashed line) and the spectra acquired in the flame with comparable composition to illustrate the spectral dampening due to inhomogeneity of the sample.

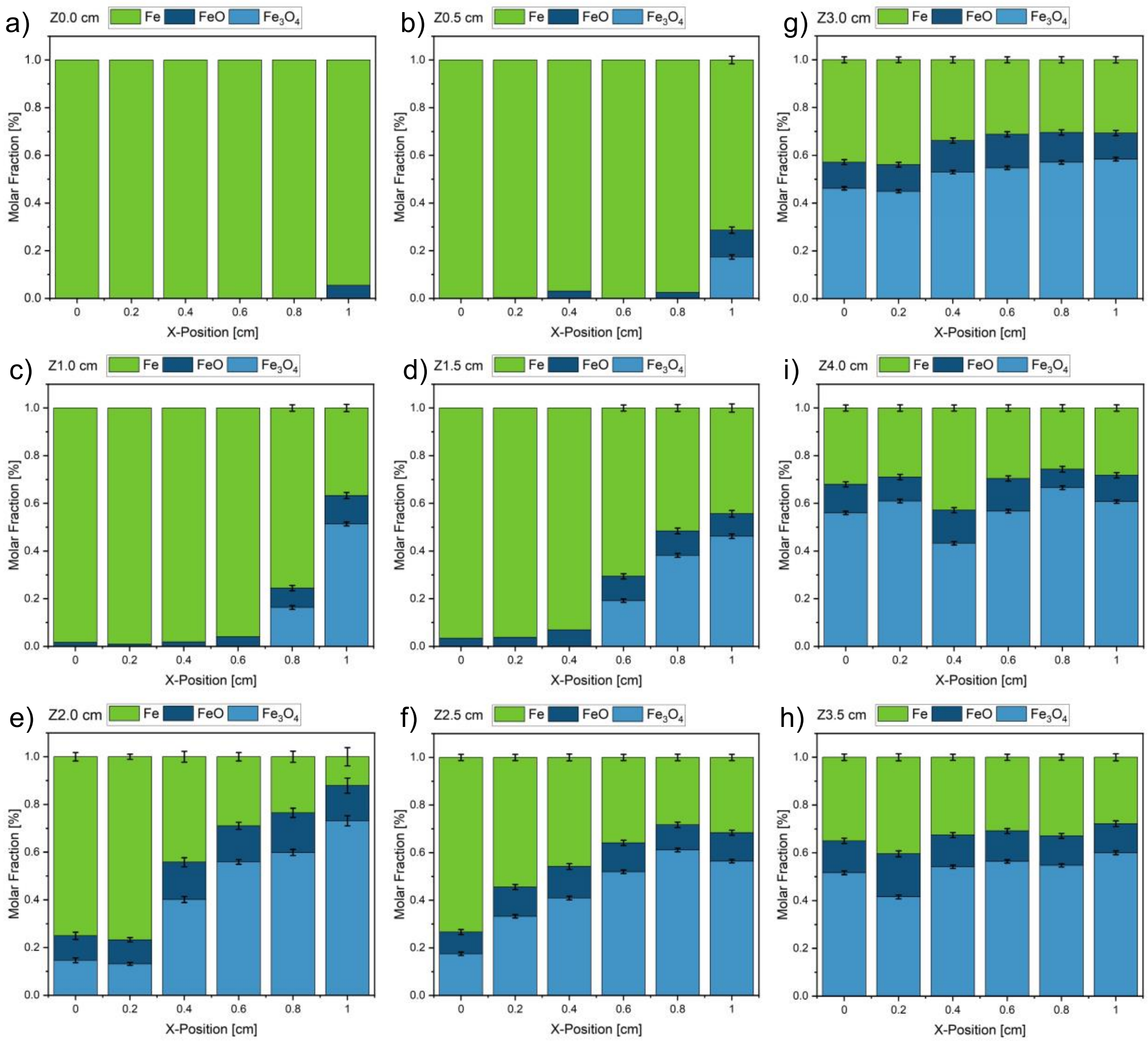

Figure S3 LCA results with three references: a) at Z=0.0cm and X=0.0 cm, b) at Z=0.5 cm and X=0.0 cm, c) at Z=1.0cm and X=0.0 cm , d) at Z=1.5cm and X=0.0 cm, e) at Z=2.0cm and X=0.0 cm, f) at Z=2.5cm and X=0.0 cm, g) at Z=3.0cm and X=0.0 cm, h) at Z=3.5cm and X=0.0 cm, i) at Z=4.0cm and X=0.0 cm
